# From wrist to heart: clinical validation and real-world translation of wearable and portable AI-enhanced electrocardiography

**DOI:** 10.1093/ehjdh/ztag155

**Published:** 2026-10-01

**Authors:** Ahmed El-Medany, Aidan René Birdi, Aron Abraham, Tanush Pandey, Arunashis Sau, Sadia Khan, Fu Siong Ng

**Affiliations:** National Heart and Lung Institute, Imperial College London, Hammersmith Campus, Du Cane Road, London W12 0NN, UK; Department of Cardiology, Imperial College London Healthcare NHS Trust, London W2 1NY, UK; Department of Cardiology, Chelsea and Westminster Hospital NHS Foundation Trust, London SW10 9NH, UK; National Heart and Lung Institute, Imperial College London, Hammersmith Campus, Du Cane Road, London W12 0NN, UK; National Heart and Lung Institute, Imperial College London, Hammersmith Campus, Du Cane Road, London W12 0NN, UK; National Heart and Lung Institute, Imperial College London, Hammersmith Campus, Du Cane Road, London W12 0NN, UK; National Heart and Lung Institute, Imperial College London, Hammersmith Campus, Du Cane Road, London W12 0NN, UK; Department of Cardiology, Imperial College London Healthcare NHS Trust, London W2 1NY, UK; National Heart and Lung Institute, Imperial College London, Hammersmith Campus, Du Cane Road, London W12 0NN, UK; Department of Cardiology, Chelsea and Westminster Hospital NHS Foundation Trust, London SW10 9NH, UK; National Heart and Lung Institute, Imperial College London, Hammersmith Campus, Du Cane Road, London W12 0NN, UK; Department of Cardiology, Imperial College London Healthcare NHS Trust, London W2 1NY, UK; Department of Cardiology, Chelsea and Westminster Hospital NHS Foundation Trust, London SW10 9NH, UK

**Keywords:** Wearable devices, Portable devices, Artificial intelligence, Electrocardiography, Clinical validation

## Abstract

Wearable and portable electrocardiogram (ECG) devices are shifting cardiovascular monitoring beyond clinician-directed testing, driven by consumer-led self-monitoring, remote care, and preventive cardiology. Artificial intelligence (AI) may enhance the value of these devices by supporting automated interpretation, but clinical translation remains uneven. This narrative review synthesises current evidence on AI-enhanced ECG (AI-ECG) analysis in wearable and portable systems, with emphasis on the gap between technical performance and real-world implementation. Although many advances have been developed using conventional 12-lead ECG datasets, deployment in wearable and portable devices introduces additional challenges related to device-specific signal acquisition, preprocessing, domain shift, and user-led recording. Evidence is most mature for atrial fibrillation detection, while applications across conduction disease, other arrhythmias, structural phenotypes, and prognostic assessment remain earlier in development and are supported by fewer prospective, device-specific evaluations. Across the field, retrospective and dataset-based studies predominate, with limited evidence that improved detection translates into better patient outcomes or health-system efficiency. Future research should evaluate performance using the intended device, population, and clinical pathway, and determine whether AI-ECG changes management without increasing unnecessary investigation or workload. Establishing clinical value, rather than improving classification accuracy alone, will determine the role of wearable and portable AI-ECG in routine cardiovascular care.

## Graphical Abstract

**Graphical Abstract:**
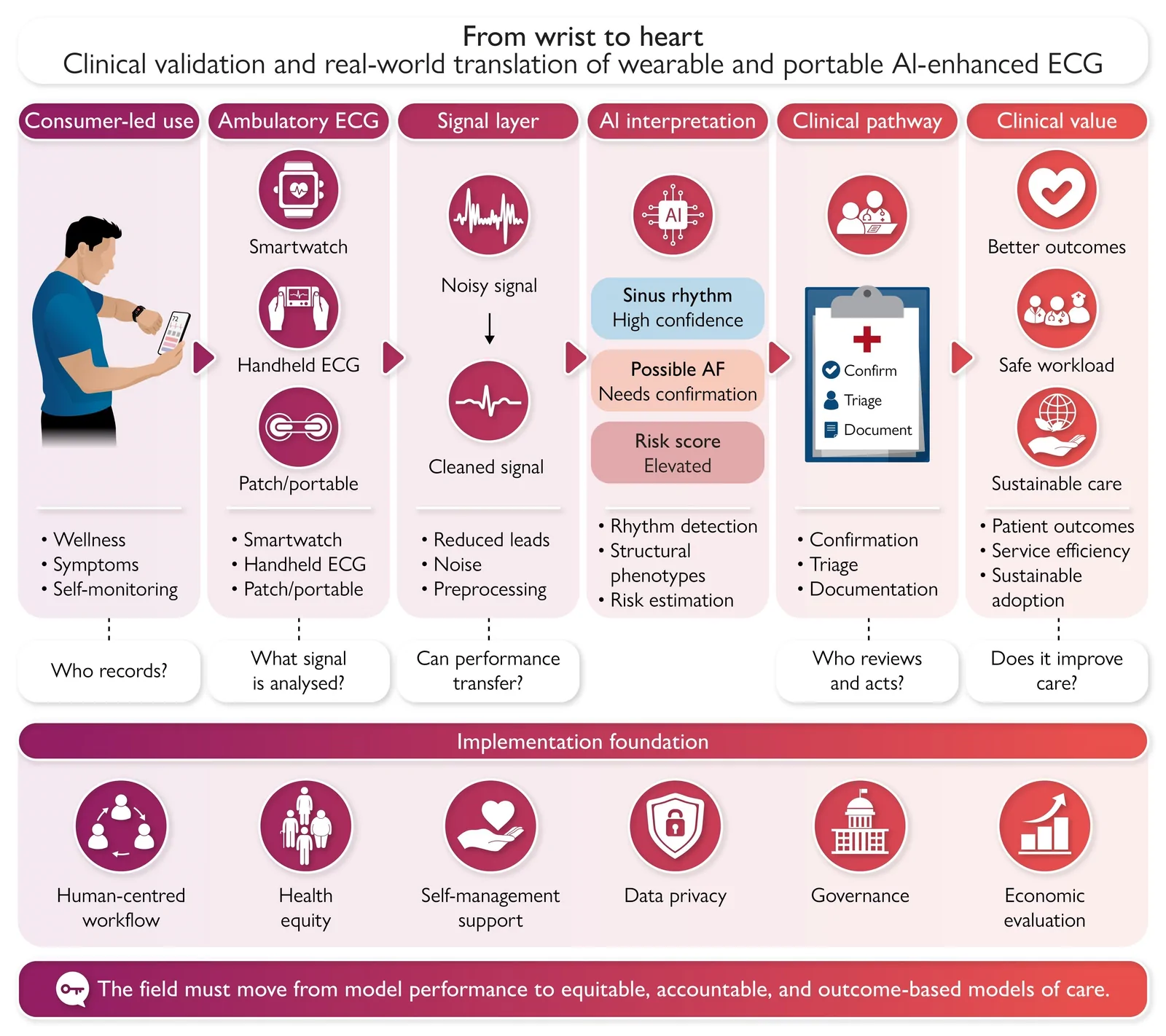

## Introduction

The electrocardiogram (ECG) is undergoing a transition from an intermittent, clinic-based investigation to a longitudinal source of patient-generated cardiovascular data. Wearable and portable ECG technologies, hereafter referred to as ambulatory ECG technologies, are central to this shift and are being adopted not only through clinician-directed remote monitoring but also through consumer use of smartwatches, handheld ECG devices, and other digital tools for wellness and self-monitoring. Wearable and portable ECG technologies therefore sit at the intersection of digital health, preventive cardiology, and routine clinical care.^1^ Although atrial fibrillation (AF), linked to 20–30% of ischaemic strokes, has dominated ambulatory ECG research, reduced-lead devices show potential across cardiomyopathies, conduction disorders, ventricular arrhythmias, and heart failure.^2–6^ Early detection is critical, as sudden cardiac death, often the first manifestation of underlying disease, accounts for nearly half of all cardiovascular deaths.^7^ Ambulatory ECG devices, including smartwatches, patches, rings, and handheld monitors, have expanded rapidly, with many incorporating lightweight artificial intelligence (AI)-based algorithms for automated interpretation in free-living environments.^8–10^

Advances in machine learning (ML) and deep learning (DL) have driven substantial improvements in ECG analysis. Convolutional neural networks (CNNs) have achieved diagnostic performance matching or exceeding expert cardiologist interpretation for arrhythmia detection.^11,12^ DL models have also been applied to artefact detection and signal pre-processing, addressing motion noise and variable signal quality inherent to wearable acquisition.^13–15^ Integrated pipelines combining acquisition, quality control, and automated interpretation now enable analyses previously requiring extensive manual review.

Despite these advances, the clinical maturity of AI-enhanced ECG (AI-ECG) remains limited across many health-system contexts, and this gap is particularly pronounced in ambulatory settings where acquisition is often user-led, signal quality is variable, and clinical responsibility for generated data may be unclear. Existing evidence remains concentrated around rhythm detection, with less mature validation for structural phenotyping, prognostic, and workflow-based applications.^16^ Many studies prioritize accuracy metrics such as sensitivity and area under the curve (AUC) without evaluating downstream clinical consequences, risking overlooked harms, including false-positive alerts, unnecessary investigations, increased clinician workload, and overtreatment.

Previous reviews have examined specific AI-ECG applications, such as AF detection, the diagnostic performance of consumer ECG wearables, and the technological and clinical use of home ECG devices.^17–19^ However, less attention has been given to the specific translational challenges that arise when AI-ECG is deployed through wearable and portable reduced-lead devices, where signal acquisition, device characteristics, user behaviour, clinical ownership, and downstream workflows differ substantially from conventional 12-lead ECG testing. This narrative review therefore follows the full ambulatory AI-ECG pathway from signal acquisition and preprocessing through clinical validation to implementation, integrating diagnostic evidence with human factors, health equity, workflow, economic, regulatory, and governance considerations. In doing so, it focuses on the gap between technical performance and real-world clinical readiness and proposes practical frameworks for assessing evidence maturity and translation across the deployment lifecycle.

### Literature identification and narrative synthesis

This narrative review was conducted as a structured synthesis of the literature on AI applied to wearable and portable ECG. A narrative approach was selected because of substantial heterogeneity across devices, signal acquisition methods, analytical pipelines, model architectures, and reported clinical and operational endpoints. The aim was not to rank individual algorithms or devices but to evaluate technical innovation, clinical validation, and real-world translational readiness.

Relevant literature was identified through searches of PubMed and Semantic Scholar, supplemented by citation and reference-list searching of key primary studies and previous reviews. The initial literature search was undertaken in February 2026 and subsequently updated during manuscript development. Search concepts encompassed artificial intelligence, ML and DL in combination with electrocardiography, wearable, portable, smartwatch, handheld, and reduced-lead devices, together with terms relating to diagnostic and prognostic applications, clinical validation, and implementation. During the initial scoping stage, Undermind was used as an AI-assisted literature-discovery tool to identify potentially relevant studies and additional search directions, consistent with emerging approaches using semantic-search tools to supplement conventional literature searching. All candidate studies were independently reviewed, verified, selected, and interpreted by the authors.^20,21^

Given the heterogeneity of study designs, formal risk-of-bias scoring was not applied. Instead, evidence was appraised qualitatively with consideration of cohort size and selection, reference standard, validation strategy, device and signal configuration, and relevance to the intended clinical setting. Greater weight was placed on independent or prospective validation using the intended wearable or portable device and clinically representative populations. Findings based predominantly on retrospective datasets, simulated reduced leads, selected or enriched cohorts, or limited independent validation were interpreted more cautiously.

For the purposes of this narrative synthesis, evidence was considered ‘established’ where findings were supported by multiple studies with independent or prospective validation in the intended device, population, and use context; ‘emerging’ where findings were promising but independent, prospective, device-specific, or real-world validation remained limited; and ‘exploratory’ where evidence was predominantly proof-of-concept, retrospective, based on simulated leads, or derived from selected cohorts without demonstrated clinical utility. These categories were used as qualitative descriptors rather than formal evidence grades.

## Technical innovation in ambulatory AI-ECG

### Signal acquisition and data characteristics

The effectiveness of wearable and portable ECG monitoring is fundamentally constrained by signal quality. In free-living environments, motion artefact, variable electrode contact, and environmental interference introduce noise that can impair rhythm interpretation and increase false-positive detection rates.^14,15^ As ambulatory ECG monitoring moves beyond controlled settings, robust signal pre-processing becomes a prerequisite for reliable downstream AI analysis. The key stages of the ambulatory AI-ECG pipeline, from acquisition to clinical action, are summarized in *Figure 1*.

**Figure 1:**
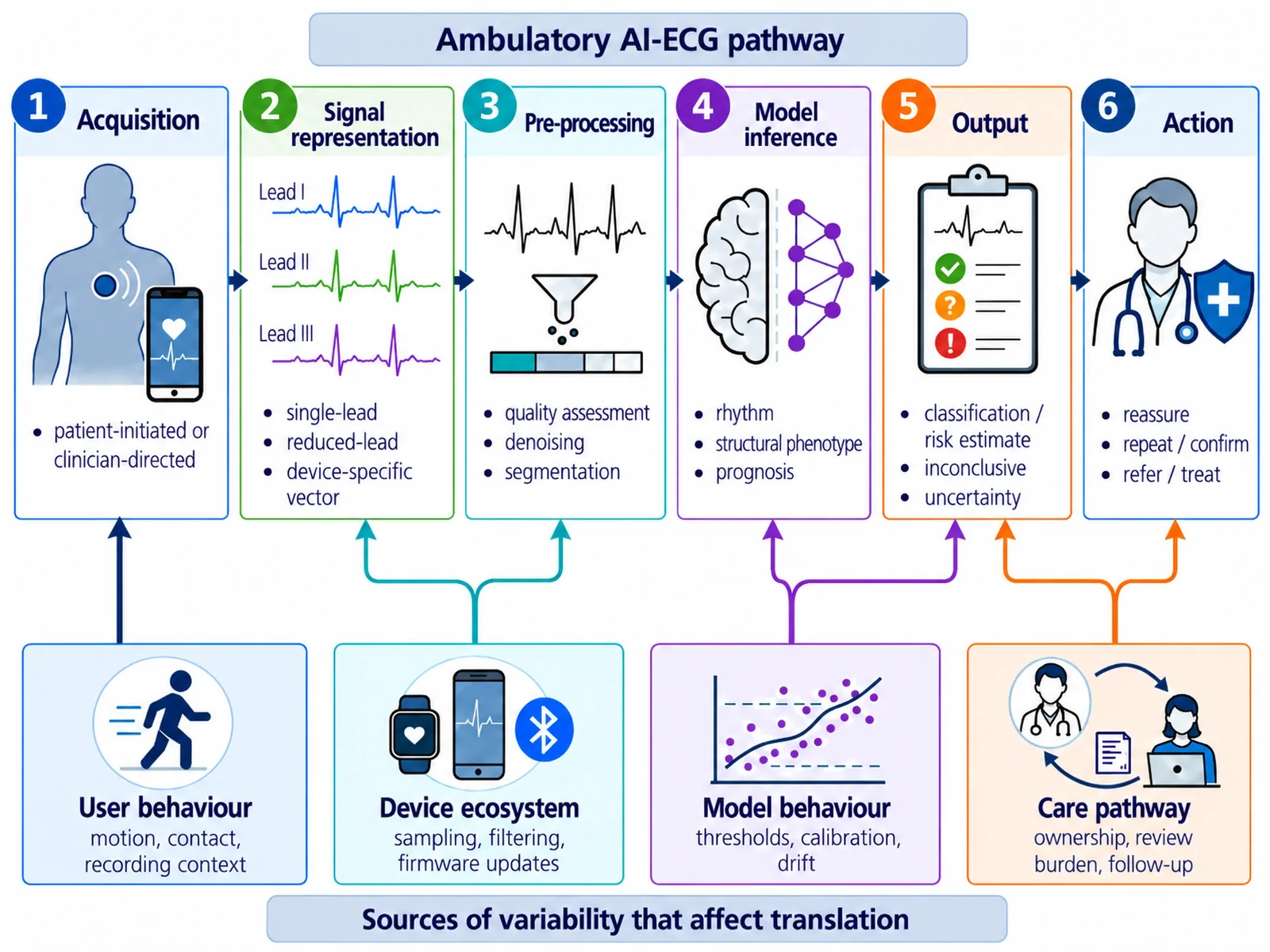
**The ambulatory AI-ECG pipeline from acquisition to action.** Wearable and portable ECG recordings move through device-specific acquisition, preprocessing, and AI interpretation before entering clinical pathways. Variability at each stage, from signal quality to clinical response, influences whether algorithmic outputs translate into meaningful care. AI-ECG, artificial intelligence-enhanced electrocardiography.

### Pre-processing and quality assessment

DL-based denoising and feature extraction techniques have demonstrated improvement in ECG signal robustness under ambulatory conditions. For example, Reljin *et al*. (2020) applied convolutional encoder–decoder architectures to armband-acquired ECG signals to denoise QRS complexes, with substantial improvement in R-peak detection accuracy (91.86% vs. 61.16%).^9^ Similarly, U-Net-based DL models trained for R-wave localization have achieved high sensitivity in noisy wearable-style datasets. Yang *et al*. (2024) trained their model on the China Physiological Signal Challenge 2019 database and subsequently demonstrated a sensitivity of 99.88% on the MIT-BIH Arrhythmia Database.^22^ These approaches illustrate the capacity of modern AI pipelines to compensate, at least partially, for the intrinsic limitations of reduced-lead and motion-affected recordings. However, most pre-processing studies report signal-level metrics rather than clinical endpoints. Whether improved waveform fidelity reduces false alerts, review burden, or changes in management remains uncertain.

### Feature learning and classification architectures

Beyond pre-processing, advances in model architecture have enabled increasingly accurate rhythm classification from reduced-lead wearable ECGs. CNNs and related DL approaches have demonstrated high discriminative performance for arrhythmia detection across large and heterogeneous ECG datasets, in some cases approaching expert cardiologist performance in externally evaluated settings.^11,12,23,24^ Subsequent developments have enabled integrated analytical pipelines combining artefact suppression, rhythm classification, and confidence estimation to support scalable screening workflows.^25^ Lightweight architectures capable of real-time or near-real-time inference under wearable hardware constraints further demonstrate the feasibility of continuous and longitudinal monitoring in consumer devices.^10^

Nevertheless, the type of validation should be clearly distinguished. External validation refers to evaluation in data independent of those used for model development, ideally drawn from a different institution, population, or setting. Cross-dataset validation can therefore provide evidence of generalisability but does not necessarily establish cross-device transportability when acquisition hardware remains similar or wearable signals are simulated from conventional ECGs. Conversely, direct cross-device evaluation tests performance on signals acquired using different hardware and may reveal additional domain shift related to lead configuration, signal processing, and device characteristics. Even successful external or cross-device validation does not guarantee consistent performance across subsequent firmware updates, hardware generations, or diverse real-world usage patterns.

### Explainability and trustworthy model design

Although diagnostic accuracy has dominated wearable ECG research, regulatory translation requires explainability and trustworthiness. *Post hoc* explainability techniques, including gradient-based activation maps and saliency methods, have been applied after model training to identify waveform regions associated with individual predictions, frequently highlighting QRS complexes, *P*-wave morphology, or rhythm irregularity.^26^ However, these methods do not make the underlying model intrinsically interpretable, and saliency maps may not reliably reflect internal decision processes and can be sensitive to noise.

In contrast, intrinsically interpretable approaches incorporate mechanisms for separating or exposing clinically meaningful features within the model architecture itself. Hammer *et al*. (2024) describe an explainable architecture (xECGArch) employing parallel CNNs to disentangle rhythmic and morphological ECG features.^24^ Their analysis suggests that the rhythm branch predominantly concentrates on QRS complexes, while the morphology branch emphasizes fibrillatory waves during AF classification. Such architectures aim to reduce reliance on opaque ‘black-box’ inference and align model outputs more closely with clinically interpretable ECG features. However, intrinsic interpretability may involve a trade-off with predictive performance; recent work in AI-ECG has demonstrated that imposing greater model explainability can reduce downstream performance.^27^

Despite these advances, explainability remains inconsistently implemented. Few primary studies incorporate formal interpretability analyses, and even fewer evaluate whether highlighted waveform regions consistently correspond to clinically meaningful ECG features.^24^ Evaluation within specific demographic groups has been reported in selected contexts, including studies focusing on older adults,^28^ but systematic assessment of performance variability across demographic subgroups remains uncommon. This gap is concerning, as algorithmic performance may vary across underrepresented populations in the absence of predefined subgroup evaluation and transparent reporting.^26^ High diagnostic accuracy and partial explainability do not guarantee reliable performance across diverse patient populations. Without consistent subgroup assessment and clear reporting standards, wearable ECG algorithms risk appearing robust in controlled validation settings yet insufficiently generalizable in real-world clinical deployment.

The field has progressed from early proof-of-concept demonstrations of DL performance^16^ to increasingly sophisticated analytical pipelines^26^ extending beyond AF to other rhythm and structural phenotypes.^23,29^ Yet retrospective accuracy alone does not establish clinical value. Prospective evaluation is required to determine real-world scalability, workflow integration, and impact on patient outcomes.

## The ambulatory AI-ECG clinical validation landscape

### Atrial fibrillation detection

AF detection remains the most extensively studied indication in wearable and portable AI-ECG research. Multiple primary studies and meta-analyses have demonstrated high diagnostic performance using smartwatch and other single-lead ECG recordings.^5,17,24–26,28–32^ In a 2025 systematic review and meta-analysis of smartwatch single-lead ECGs across multiple manufacturers, Iqhrammullah *et al*. (2025) reported a summary area under the receiver operating characteristic curve (AUC) of 0.95 (95% confidence interval [CI] 0.93–0.97) for algorithmic AF detection, with pooled sensitivity of 86% (95% CI 80–91%) and specificity of 94% (95% CI 89–97%).^30^ However, substantial between-study heterogeneity was present for both sensitivity (*I*^2^ = 93.3%) and specificity (*I*^2^ = 95.8%), reflecting variability across devices, populations, and study designs and limiting direct extrapolation of the pooled estimates to individual devices or real-world screening populations. High discrimination has also been reported in broader ECG-based AI literature. Xie *et al*. (2024) reported an AUC of 1.00 (95% CI 0.99–1.00) for deep-learning approaches compared with 0.98 (95% CI 0.97–0.99) for traditional machine-learning methods.^31^ These near-ceiling estimates require particular caution: the meta-analysis was not restricted to wearable ECG, included studies based predominantly on controlled or public ECG datasets, and demonstrated substantial between-study heterogeneity. Such estimates may therefore overstate expected performance when algorithms are deployed in lower-prevalence, user-acquired, real-world wearable settings.

Beyond contemporaneous AF detection, AI models have increasingly explored prediction of future or paroxysmal AF from reduced-lead signals. However, the signal source is important when judging translational readiness. For example, Choi *et al*. (2025) developed a lead-I model for prediction of new-onset AF using lead I extracted retrospectively from standard 12-lead ECGs, rather than ECGs acquired directly using a wearable device.^32^ Although such studies demonstrate that predictive information may be preserved in reduced-lead representations, they should be regarded as proof-of-concept evidence for wearable deployment until validated using the intended wearable or portable hardware and acquisition environment.

### Beyond atrial fibrillation detection

While AF detection has reached relative diagnostic maturity in ambulatory AI-ECG research, emerging studies suggest broader diagnostic potential across arrhythmic and conduction phenotypes. Earlier proof-of-concept studies using benchmark ambulatory ECG datasets demonstrated that DL models could classify supraventricular and ventricular ectopy, fusion beats, and other rhythm classes.^33–36^ For example, Acharya *et al*. (2017) developed a CNN using lead-II recordings from the MIT-BIH Arrhythmia Database to classify five heartbeat categories, while Romdhane *et al*. (2020) subsequently evaluated a 1D-CNN using the MIT-BIH and INCART datasets and reported an overall accuracy of 98.41%.^35,36^ However, these were retrospective, dataset-based evaluations rather than validation using contemporary wearable or portable ECG devices, limiting direct extrapolation to real-world ambulatory use.

More directly relevant evidence has emerged from portable single-lead ECG systems. Sangha *et al*. (2024) developed a multilabel CNN using synthetic lead-I representations derived from more than 3 million clinical 12-lead ECGs and subsequently evaluated the model on 24 808 cardiologist-annotated single-lead ECGs acquired using the AliveCor KardiaMobile.^5^ In this independent portable-device dataset, AUCs were 0.95 for premature ventricular contractions, 0.91 for premature atrial contractions, 0.94 for first-degree atrioventricular block, and 0.99 for second- or third-degree atrioventricular block.^5^ The model also showed high discrimination for AF/flutter, supraventricular tachycardia, wide-QRS rhythms, and paced ECGs. However, this evaluation was retrospective and is currently reported as a conference abstract, so prospective validation in representative populations and clinical pathways remains required.

Emerging work has also explored structural heart disease phenotyping from reduced-lead ECG signals, particularly for the detection of left ventricular systolic dysfunction (LVSD). Such models aim to extract latent structural information from interval and waveform patterns, extending ambulatory AI-ECG beyond rhythm classification. Early proof-of-concept studies demonstrated that AI applied to lead I retrospectively extracted from standard 12-lead ECGs could identify LVSD. Cho *et al*. (2021) reported single-lead AUCs of 0.874 (95% CI 0.859–0.890) and 0.929 (95% CI 0.911–0.946) in internal and external hospital datasets, respectively, although these signals were derived from conventional 12-lead ECGs rather than acquired using a wearable device.^37^ Subsequent studies have evaluated models using ECGs acquired directly from consumer devices. Kwon *et al*. (2022) assessed asynchronously acquired lead-I and lead-II ECGs from Samsung Galaxy Watch Active and Apple Watch Series 6 devices in 755 patients, reporting an overall AUC of 0.934 (95% CI 0.913–0.955) for left ventricular ejection fraction (LVEF) ≤ 40%. Despite high negative predictive value (NPV), positive predictive values were ∼0.22–0.23, illustrating the importance of prevalence and downstream confirmatory testing when considering screening applications.^23^ Attia *et al*. (2022) subsequently evaluated single-lead Apple Watch ECGs acquired remotely by patients in non-clinical environments, achieving an AUC of 0.885 (95% CI 0.823–0.946) for LVEF ≤40%. However, only 421 participants had an eligible watch ECG within 30 days of echocardiography, and only 16 had LVSD, resulting in relatively wide CIs despite the prospective, decentralized design.^38^

Point-of-care portable devices provide a complementary pathway for reduced-lead AI-ECG screening. In a prospective multicentre study of 1050 patients, Bachtiger *et al*. (2022) applied AI to single-lead ECGs acquired using the Eko DUO ECG-enabled digital stethoscope, reporting an AUC of 0.85 (95% CI 0.81–0.89) from the best-performing single recording position; an exploratory model combining outputs from two recording positions achieved an AUC of 0.91 (95% CI 0.88–0.95).^39^ More recently, Guo *et al*. evaluated an Eko DUO digital-stethoscope model incorporating both single-lead ECG and phonocardiogram signals in 2960 patients across four healthcare networks, reporting an AUC of 0.85, sensitivity of 77.5%, specificity of 78.3%, and NPV of 98.0% for LVEF ≤40%.^40^

Collectively, these studies support the feasibility of detecting LVSD from reduced-lead ECG signals, including recordings acquired directly from consumer and point-of-care devices. However, performance varies across acquisition systems and study designs, and much of the evidence remains based on selected populations undergoing echocardiography or relatively small numbers of patients with reduced LVEF. Demonstrating consistent performance in representative screening populations, together with evidence that detection leads to appropriate confirmatory testing and treatment, remains necessary before LVSD screening using ambulatory AI-ECG can be considered clinically established.

### Emerging and future applications of ambulatory AI-ECG

Beyond established rhythm applications, reduced-lead AI-ECG has been explored for physiological monitoring and cardiovascular risk prediction. Alam *et al.* (2024) developed QTNet using lead-I ECGs to estimate QT intervals, achieving mean absolute errors of 12.63 ms and 12.30 ms in internal and external validation datasets, respectively. When subsequently applied to detection of dofetilide-induced QTc prolongation, the model achieved 87% sensitivity and 77% specificity.^41^ However, these evaluations used high-quality clinical lead-I signals rather than ECGs acquired directly from ambulatory devices, and the authors acknowledged that performance may not directly translate to noisier wrist-worn recordings.

Proof-of-concept studies have similarly explored systemic cardiovascular phenotypes. In a prospective two-centre cohort of 1254 individuals without overt cardiovascular disease, Angelaki *et al*. (2025) applied a machine-learning model to lead I isolated from conventional 12-lead ECGs, achieving an AUC of 0.831 (95% CI 0.781–0.871), with 72% sensitivity and 82% specificity for distinguishing hypertensive from normotensive individuals.^42^ Although prospectively collected, the ECGs were acquired using a clinical digital electrocardiograph rather than a wearable device, and the authors accordingly described wearable translation as requiring further validation. Feature importance analysis in this study highlighted contributions from both conventional risk factors (age and body mass index) and ECG-derived morphological characteristics, including QRS and T-wave features, supporting the concept that ECG signals encode latent haemodynamic and vascular information beyond traditional electrophysiological interpretation.

AI-ECG has also shown potential for prognostic assessment. Sau *et al*. (2024) developed a lead-I-only version of the AIRE model (AIRE-1L), which predicted all-cause mortality with a C-index of 0.751 (95% CI 0.750–0.752), only modestly lower than the multilead model.^43^ However, AIRE-1L was evaluated using lead I from conventional ECG datasets rather than ECGs acquired directly from wearable devices, and therefore demonstrates preservation of prognostic information in a reduced-lead representation rather than validation of a wearable prognostic tool.

Importantly, recent direct cross-device evaluation reinforces this distinction. Lin *et al*. (2026) found that lead-I models trained on conventional clinical ECGs showed relatively little degradation during cross-hospital validation (AUC decline <0.03), but substantially greater performance loss when transferred directly to Apple Watch and QOCA ECG102D single-lead recordings. For low-EF detection, for example, AUC decreased from approximately 0.88 in the clinical test dataset to 0.82 with Apple Watch and 0.75 with QOCA ECG102D recordings; device-specific fine-tuning subsequently improved performance to 0.85 and 0.86, respectively.^44^ These findings demonstrate that preservation of predictive information within lead I does not itself establish transportability across acquisition devices. Consequently, broader physiological and prognostic applications of ambulatory AI-ECG should currently be considered exploratory, pending device-specific validation, prospective evaluation in intended populations, and demonstration that risk predictions lead to actionable changes in care.

## Workflow integration and real-world implementation

Despite strong diagnostic performance, workflow integration and cost-effectiveness remain rarely addressed in ambulatory AI-ECG studies. Systematic reviews consistently identify these domains as major evidence gaps.^17,18^ The practical performance of wearable and portable AI-ECG systems, therefore, depends not only on algorithmic accuracy but on usability, clinical workflow compatibility, and economic sustainability.

### User interaction and data acquisition

Structured usability data remains limited. One of the few real-world evaluations, the Samsung Simband 2 smartwatch study, utilized a multimodal platform capable of PPG and single-lead ECG acquisition and demonstrated acceptable usability and comfort in older adults during daily activity.^28^ Across devices, signal quality is highly sensitive to user behaviour: motion artefacts, sweating, and suboptimal electrode placement frequently degrade ECG recordings in wearable studies.^9,13,45^ These acquisition challenges directly affect diagnostic reliability and increase downstream review burden.

AI-based quality control and denoising pipelines partially mitigate this problem. CNN-based filtering systems for handheld and portable ECGs reduce invalid segments and lower false AF alerts by ∼22–25%.^13,45^ Usability challenges also extend to the cognitive demands placed on users. However, the downstream impact on clinician review time and triage workload is rarely quantified, and improvements in sensitivity may paradoxically increase notification burden by detecting clinically insignificant abnormalities. User factors further influence effectiveness. Reviews consistently highlight barriers, including digital literacy, device operation, and sustained engagement for reliable data capture.^17^ Inconsistent acquisition produces incomplete or unusable ECG traces, undermining both diagnostic performance and workflow efficiency.^17–19^ These user-facing challenges also have implications beyond signal quality. As ambulatory ECG technologies move into consumer-led use, their effectiveness depends on who can access and use them reliably, and how patient-generated outputs are analysed and acted upon.

### Health equity, consumer-led use, and self-management

Smartwatches and handheld devices are often used for wellness and self-monitoring before they enter formal healthcare pathways. Large-scale consumer studies, including the Apple Heart Study and Fitbit Heart Study, demonstrate how photoplethysmography (PPG)-based consumer wearables can identify possible AF and trigger confirmatory ECG monitoring outside conventional screening programmes.^46,47^ This consumer-led model creates opportunities for earlier recognition of CVD and more active patient involvement in care but also raises questions about access and the interpretation of patient-generated outputs.^48^

Equity considerations should feature at all stages of the ambulatory AI-ECG pipeline: dataset curation, model development, evaluation, deployment, and post-deployment monitoring.^49^ Large clinical training datasets may not fully represent populations using consumer wearable and portable devices, where indications for recording, disease prevalence, and healthcare priorities differ. Models achieving high performance in high-prevalence clinical cohorts may not retain meaningful clinical utility in healthier populations, where pre-test probability is substantially lower.

The demographics of wearable use compound these problems. Consumer device ownership in the USA is concentrated among people with higher household incomes, higher educational attainment, and younger age,^50^ and wearables remain under-researched in low- and middle-income countries.^51^ The populations who may benefit most from this technology, those who are older and socioeconomically disadvantaged, may therefore be systematically underrepresented in both wearable device use and the datasets in which these devices are validated.

Investigation of algorithmic bias in wearable and portable AI-ECG tools is limited. Twelve-lead AI-ECG algorithms can learn and may rely on race-correlated features,^52,53^ a finding that is likely to extend to single-lead wearable signals. As standardized reporting frameworks for model development, validation, and deployment have matured,^54–56^ the reporting of subgroup performance has become a baseline expectation. However, the AI-ECG bias literature remains sparse: a recent scoping review protocol identified few eligible studies reporting subgroup performance or fairness metrics and noted that existing reviews of AI-ECG seldom report performance stratified by demographic or geographic subgroups.^49^

Performance inequity is not necessarily one-dimensional. A model may discriminate equally well across subgroups while being differentially miscalibrated, so fixed clinical thresholds can lead to differential referral patterns, treatment decisions, or reassurance across populations.^57,58^ Future studies should therefore evaluate calibration, usability, access to confirmatory testing, and downstream clinical consequences across relevant demographic, socioeconomic, and geographic groups. In addition, post-deployment monitoring across patient subgroups will be necessary to prevent exacerbating existing care disparities and ensure fair implementation.^17,59^

Self-management adds a further layer of complexity. Consumer-facing outputs may be interpreted as medical advice, even when intended as screening or wellness information. False-positive alerts may increase health anxiety and unnecessary investigation, while false-negative outputs may provide inappropriate reassurance. To support safe self-management, ambulatory AI-ECG systems require clear user-facing information, defined escalation routes, and integration with clinical services capable of confirming diagnoses and contextualizing risk. The goal should not be to shift clinical responsibility onto patients but to develop supported models of care in which patient-generated ECG data can contribute to prevention, monitoring, and shared decision-making.^60^

### Clinical workflow integration

Clinical workflow integration is particularly challenging as many ambulatory ECG recordings are currently generated outside clinician-directed pathways. Patient-initiated recordings may therefore enter healthcare systems through various channels, but responsibility for reviewing and acting on these data remains poorly defined. Most prospective and real-world studies do not specify who is responsible for interpreting AI-ECG outputs: whether primary care clinicians, cardiologists, or remote monitoring services, nor how frequently such data should be assessed, and clinician responses to alerts are rarely characterized in practice.^61^

Interoperability presents an additional barrier. Wearable ECG recordings are frequently exported as PDFs, with limited integration into electronic health record systems due to a lack of interoperability standards (e.g. HL7/FHIR-compatible data transfer).^5,19,62^ As a result, monitoring often relies on manual uploads or email-based transfer, fragmenting care processes and restricting scalability. Even when diagnostic performance generalizes across hardware platforms,^63,64^ workflow integration remains device-specific, and even high-performing models cannot scale if clinical systems cannot ingest, triage, or store data efficiently. Proposed service models, including telehealth rhythm hubs, nurse-led triage, and AI-first filtering, have been suggested but not prospectively evaluated, leaving their effect on clinician workload uncertain.

### Alert burden and actionability

A major unresolved barrier to ambulatory AI-ECG implementation is alert burden. Although this has been most extensively quantified in implantable device monitoring, wearable and portable device studies already show early signals of the same problem. In the Apple Heart Study, irregular pulse notifications were uncommon, but more than half of notified participants contacted a healthcare provider and over one-third were advised to undergo further testing.^47^ In a real-world Mayo Clinic evaluation of patients presenting after PPG-based Apple Watch abnormal pulse detection, most underwent diagnostic testing, yet a clinically actionable cardiovascular diagnosis was identified in only a minority.^65^ False smartwatch AF alerts have also been associated with reduced perceived physical well-being and lower confidence in chronic symptom management,^66^ while wearable use among patients with established AF has been linked to greater low-yield symptom and rhythm monitoring, treatment concern, and clinician contact.^67^

The remote-monitoring literature from implantable loop recorders (ILRs) and cardiac implantable electronic devices (CIEDs) provides a further warning about what can happen when cardiac monitoring becomes more continuous and systematized. In large CIED cohorts, remote monitoring generates substantial alert volumes, with ILRs contributing disproportionately to transmissions despite representing a minority of devices.^68^ False-positive ILR alerts are common and are frequently related to AF, pause, or bradycardia detections caused by ectopy, sensing errors, signal dropout, noise, or device programming.^69–71^ Although ILRs are prescribed to selected patients and embedded within device-clinic infrastructure, this experience is relevant to consumer wearable and portable AI-ECG because patient-initiated recordings could generate even larger volumes of data without equivalent clinical pathways.

Mitigating alert burden will require more than improving algorithmic specificity. Experience from ILR and remote device-clinic settings suggests that workload falls when alerts are filtered and prioritized before reaching clinicians, and when the clinical response to each alert type is clearly defined.^72–77^ This principle is likely to be relevant to wearable and portable AI-ECG, although it has not yet been adequately tested in consumer-led pathways. The recording should not simply be classified as abnormal; it should be placed in context, assigned a level of confidence, and routed according to whether it is likely to require clinical action.

Future studies should evaluate ambulatory AI-ECG as a service intervention, not a diagnostic classifier alone. The key question is whether alerts enable timely care or instead generate unnecessary review, over-investigation, reassurance-driven consultations, and anxiety. Without this evidence, technically accurate systems may still increase workload, uncertainty, and low-value care.

### Economic considerations

Health economic evidence is similarly limited. Hardware, software subscriptions, and cloud storage all contribute to cost, yet these elements are rarely quantified. Consumer-led monitoring may also shift costs into health systems indirectly, as patient-generated ECGs and device notifications create demand for clinical review, confirmatory testing, and reassurance. One of the few comprehensive evaluations, a microsimulation decision-analytic model by Chen *et al*. (2022), examined AF screening strategies across a simulated population of 30 million individuals aged ≥65 years. Screening using wrist-worn wearable devices was associated with gains of 226–957 quality-adjusted life-years (QALYs) per 100 000 individuals compared with no screening, alongside reductions in stroke incidence of ∼20–23 per 100 000 person-years, albeit with an increase in major bleeding events of 20–44 per 100 000 person-years.^61^ The preferred strategy—PPG-based wearable screening followed by confirmatory wearable ECG monitoring—demonstrated an incremental cost-effectiveness ratio of approximately $57 894 per QALY, meeting conventional willingness-to-pay thresholds.^61^

Costs are highly sensitive to specificity: even modest false-positive rates trigger downstream investigations such as repeat ECGs or ambulatory monitoring. Given the prevalence of false positives in free-living environments, the economic burden of follow-up testing is likely substantial but largely unmeasured.^26,45^ Resource implications also differ across health systems; reimbursement mechanisms such as remote monitoring billing codes facilitate uptake in some settings, whereas publicly funded systems lack equivalent structures.^19,66^ Across systematic reviews, prospective cost-effectiveness evaluations for wearable AI-ECG systems are described as ‘almost absent’.^24,26^

### Pathway to implementation and sustained oversight

For ambulatory AI-ECG to progress from technical feasibility to routine care, implementation must be evaluated across the full deployment lifecycle. Prospective studies should show that algorithm-guided detection changes management and improves patient- or service-level outcomes, rather than simply increasing diagnostic activity. This requires predefined pathways for alert review, confirmatory testing, escalation, documentation, and patient-facing communication, particularly when recordings are consumer-initiated rather than clinician-prescribed. These requirements are summarized in *Table 1*.

**Table 1:** **Lifecycle framework for translating ambulatory AI-ECG into clinical care.** Key considerations before, during, and after deployment of wearable or portable AI-ECG systems within a clinical pathway

Domain	Before deployment	During deployment	After deployment
**Validation**	Match datasets to device, context, and target population	Test prospectively within predefined pathways	Track calibration, drift, subgroup performance, and clinical outcomes
**Signal and device transparency**	Define lead configuration, sampling, filtering, and signal-quality standards	Report signal quality, uncertainty, and inconclusive outputs	Reassess after device or software changes
**Clinical workflow**	Define ownership, escalation, confirmation, and actionable-alert criteria	Quantify alert burden, review time, referrals, and downstream testing	Refine pathways using safety, workload, and outcome data
**Human factors and self-management**	Provide accessible user information and realistic expectations	Assess understanding, anxiety, adherence, and escalation behaviour	Ensure self-management remains supported
**Health equity**	Ensure representative populations and deployment settings	Assess access, usability, calibration, and subgroup performance	Monitor disparities in referral, reassurance, and treatment
**Governance and privacy**	Establish consent, cybersecurity, auditability, and accountability	Integrate patient-generated ECG data securely into clinical systems	Update governance as models and pathways evolve
**Economic and service evaluation**	Estimate device, software, review, storage, and testing costs	Measure workload, resource use, and opportunity costs	Determine cost-effectiveness and service-level benefit

AI-ECG, artificial intelligence-enhanced electrocardiography.

Progression from promising diagnostic performance to clinical validation should require prospective evaluation in the intended device, population, and care pathway. Beyond diagnostic yield, studies should establish whether AI-ECG changes management or accelerates diagnosis and treatment without generating disproportionate false-positive alerts, downstream investigation, or clinician workload. Prospective diagnostic, implementation, and pragmatic comparative studies will therefore be required to move applications from exploratory or emerging evidence towards clinically validated use (*Figure 2*; *Table 1*).

**Figure 2:**
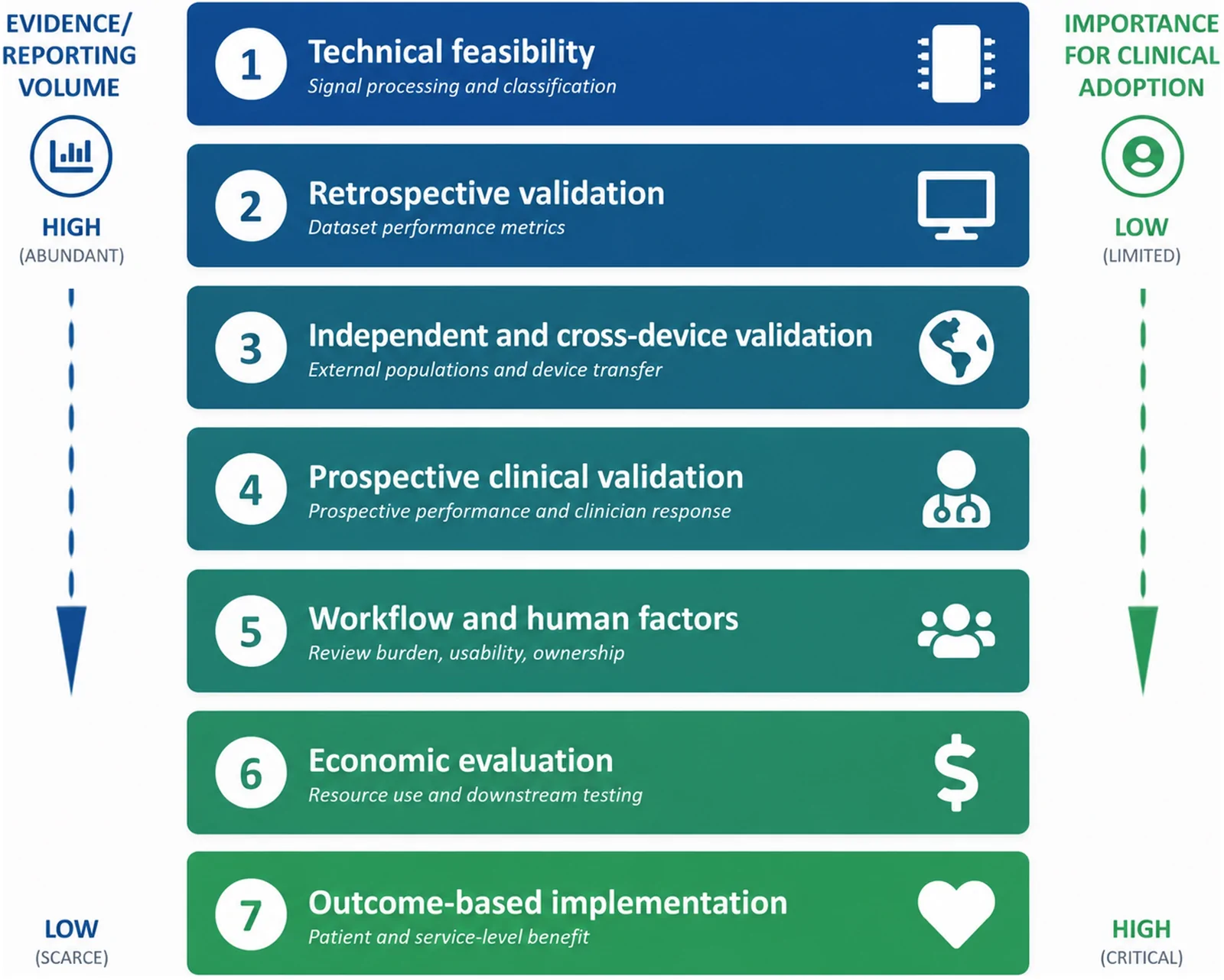
**The evidence maturity gap in wearable and portable AI-ECG.** Current evidence is strongest for technical feasibility and retrospective diagnostic validation, but less mature for prospective workflow, economic, and outcome-based evaluation. Translation into routine care will require moving beyond accuracy metrics towards real-world evidence of clinical value. AI-ECG, artificial intelligence-enhanced electrocardiography.

Secure integration with clinical information systems is necessary to ensure traceability, documentation, and accountability. Interoperable data transfer and clear audit trails allow wearable- and portable-generated ECGs to become part of the clinical record rather than remaining external data streams.^18^ Without this infrastructure, large-scale deployment risks increasing administrative workload rather than improving care efficiency.

An additional practical consideration is the representation of ECG signals across devices. Wearable and portable systems frequently record non-standard lead configurations (e.g. lead I equivalents or device-specific vectors), which differ from the spatial information captured in conventional 12-lead ECGs. Models trained on standard 12-lead datasets may therefore exhibit reduced or unpredictable performance when applied to wearable signals due to differences in lead orientation, signal preprocessing, and noise characteristics. This domain shift highlights the importance of training and validating models on data that reflect the intended deployment environment, including device-specific signal representations. Approaches such as lead-specific modelling, domain adaptation, and training on heterogeneous multi-device datasets may improve generalisability but remain incompletely evaluated in prospective settings.^12,46,78^

Regulatory approval, reimbursement, and governance must also align with sustainable adoption. This includes monitoring performance over time, managing software updates, defining responsibility for alerts, and ensuring usability across real-world populations.^27,28^ Adoption therefore depends not only on demonstrating accuracy, but also on sustaining reliability, accountability, and operational practicality after deployment.

## Regulation, governance, and ethical oversight

The translation of AI-ECG wearable and portable technologies into routine clinical settings relies on both technical performance and on regulatory and governance frameworks that ensure safe and accountable deployment. As these systems evolve from investigational tools to clinical instruments, they increasingly fall under medical device regulation.

In the United States, the Food and Drug Administration (FDA) increasingly applies a total-product-lifecycle approach to AI-enabled medical devices, with emphasis on clinical validation, transparency, and ongoing performance assessment. Predetermined Change Control Plans can prospectively specify certain future modifications together with the methods used to develop, validate, and assess their impact.^79,80^ Similar expectations apply in Europe under the Medical Device Regulation (MDR), while AI-enabled medical devices may additionally fall within the high-risk provisions of the European Union Artificial Intelligence Act, depending on their regulatory classification and conformity-assessment requirements.^81^ Together, these frameworks extend evaluation beyond initial diagnostic accuracy towards safety, accountability, and performance throughout the device lifecycle.

A further challenge is that regulatory validation of AI-ECG may be linked to the hardware through which the ECG is acquired. Although an algorithm may theoretically be transferable between devices, its clinical performance can depend on the lead vector, electrodes, sampling frequency, filtering, firmware, and preprocessing of the acquisition system. Regulatory evaluation may therefore apply to a defined software–hardware configuration rather than to the algorithm in isolation, restricting transfer of otherwise well-performing models between commercial platforms and potentially requiring additional validation following material hardware or software changes.^82^ This also creates dependence on the commercial lifecycle of the paired device, as discontinuation, substantial updates, or loss of manufacturer support may affect continued deployment even when the underlying algorithm remains technically viable.

Consumer ECG acquisition amplifies these governance challenges. Unlike clinician-requested investigations, ambulatory AI-ECG recordings may be initiated by patients in response to symptoms, device notifications, or reassurance-seeking before any clinical pathway has been defined. Patients may interpret algorithmic outputs as medical advice, even when intended for screening or wellness use. Governance frameworks should therefore clearly define responsibility for interpretation, escalation, and communication. Qualitative studies indicate that patients derive reassurance when clinicians understand how AI outputs are generated, describing comfort in knowing that clinicians ‘understand the machines they are dealing with.’^83^ Interpretability, therefore, functions not only as a technical property but as a clinical safety mechanism, allowing clinicians to contextualize algorithmic recommendations and challenge outputs when necessary.^84^ This supports safe disagreement between human and algorithmic assessment and enables feedback loops for system improvement.^83,84^

As discussed above, equity considerations are central to wearable and portable AI-ECG implementation. Governance frameworks should therefore require evidence that models are evaluated across relevant populations and that post-deployment monitoring can detect emerging disparities in performance, access, or downstream care.

Continuous and longitudinal data collection introduces additional privacy and cybersecurity considerations. Ambulatory devices generate longitudinal physiological data that are often transmitted via consumer communication channels, creating potential vulnerabilities. Regulatory frameworks such as the General Data Protection Regulation (GDPR) and the United Kingdom Data Protection Act mandate principles including informed consent, data minimization, and secure data handling, yet implementation varies across platforms and devices.^70^ Robust encryption, auditability, and transparent data governance processes are therefore essential to maintain patient trust and ensure safe deployment.^78,85^

Wearable and portable AI-ECG thus represents less a new diagnostic test than a new mode of cardiovascular observation. Its clinical value will depend on whether detection meaningfully alters management rather than simply increasing information. The next phase of research must prioritize outcome-based evaluation and service-level integration to determine where these systems improve care, and where they do not.

## Discussion

Across the technical, clinical, and implementation literature, a consistent pattern emerges: innovation in wearable and portable AI-ECG is advancing more rapidly than real-world clinical readiness. This evidence maturity gap is summarized in *Figure 2*. Although high discrimination has been demonstrated across several applications, much of the evidence derives from retrospective or selected cohorts, conventional ECG datasets, or reduced-lead representations rather than prospective evaluation using the intended wearable or portable device.^30,31,37,43^ Prospective evidence linking these technical gains to improved clinical management or patient outcomes remains limited.^18,19^

A central limitation lies in how generalisability is established. Performance may remain stable across clinical datasets yet deteriorate when models are transferred between acquisition systems, highlighting that cross-dataset validation does not necessarily establish cross-device transportability.^44^ Evidence remains most mature for AF detection, whereas conduction disorders, structural and systemic phenotypes, and prognostic applications are supported by fewer prospective and device-specific studies.^18,19^ More importantly, even robust diagnostic performance does not establish clinical utility without evidence that detection leads to appropriate treatment, improves outcomes, or delivers benefit without disproportionate downstream investigation and workload.

Operational evidence remains similarly underdeveloped. Factors determining practical usefulness, including patient adherence, clinician behavioural response, and healthcare workload, are rarely evaluated despite their importance for implementation.^29,46,59,62^ Integration barriers, such as limited interoperability and unclear responsibility for alert review, further constrain adoption, while governance challenges include accountability, transparency, cybersecurity, and dependence on evolving commercial hardware.^59,79–85^ These considerations extend across the entire deployment lifecycle and are summarized in *Table 1*, from device- and population-matched validation before deployment, to monitoring of workload, equity, performance drift, and clinical outcomes after implementation.

Taken together, the principal barrier is no longer signal interpretation alone, but translation into equitable, human-centred, and sustainable models of care. Moving applications from exploratory or emerging evidence towards clinically validated use will require prospective evaluation of how ambulatory AI-ECG changes clinical decision-making, self-management, workload, health-service use, and ultimately patient outcomes.

## Conclusion

Wearable and portable AI-ECG systems have advanced rapidly from experimental signal classification towards tools for consumer-led self-monitoring, screening, remote risk stratification, and longitudinal cardiovascular monitoring. Evidence is most mature for AF detection, while applications across conduction disease, structural phenotypes, and prognostic assessment remain earlier in development and are supported by fewer prospective, device-specific evaluations. Despite promising diagnostic performance, much of the literature remains retrospective or dataset-based, and convincing evidence of improved clinical outcomes remains limited. The primary challenge is therefore not detection alone, but translation into care. Uncertain clinical ownership, fragmented data integration, and limited workflow and economic evaluation continue to constrain adoption. Future studies must establish performance in the intended device, population, and clinical pathway and determine whether AI-ECG meaningfully changes management and improves patient or service-level outcomes. Establishing clinical value, rather than improving classification accuracy alone, will determine whether these technologies become part of sustainable clinical pathways.

## Lead author biography


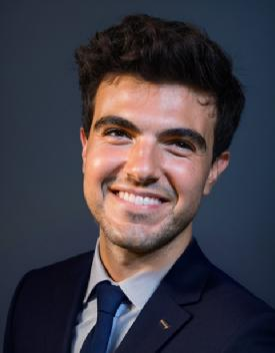


Dr Ahmed El-Medany is a senior cardiology registrar and British Heart Foundation Clinical Research Training Fellow at Imperial College London. His doctoral research focuses on artificial intelligence-enhanced electrocardiography, with particular interest in structural heart disease detection, cardiovascular risk prediction, wearable and portable ECG technologies, and the clinical implementation of AI tools in real-world healthcare systems. He works across cardiology, electrophysiology, digital health, and clinical research, with ongoing collaborations spanning academia, NHS organizations, engineering, data science, and industry. His broader academic interests include responsible AI deployment, external validation, and equitable access to cardiovascular diagnostics.

## References

[1] Jensen MT, Treskes RW, Caiani EG, Casado-Arroyo R, Cowie MR, Dilaveris P, et al ESC working group on e-cardiology position paper: use of commercially available wearable technology for heart rate and activity tracking in primary and secondary cardiovascular prevention—in collaboration with the European Heart Rhythm Association, European Association of Preventive Cardiology, Association of Cardiovascular Nursing and Allied Professionals, Patient Forum, and the Digital Health Committee. Eur Heart J Digit Health 2021;2:49–59. 10.1093/ehjdh/ztab011 PMC9753086 36711174

[2] Kim KH, Kwon JM, Pereira T, Attia ZI, Pereira NL. Artificial intelligence applied to cardiomyopathies: is it time for clinical application?. Curr Cardiol Rep 2022;24:1547–1555. 10.1007/s11886-022-01776-4 36048306

[3] Galea R, Seiffge D, Raeber L. Atrial fibrillation and ischemic stroke despite oral anticoagulation. J Clin Med 2023;12:5784. 10.3390/jcm12185784 PMC10532406 37762726

[4] Hasumi E, Fujiu K, Chen Y, Miyamoto S, Oida M, Shimizu Y, et al Heart failure monitoring with a single-lead electrocardiogram at home. Int J Cardiol 2025;432:133203. 10.1016/j.ijcard.2025.133203 40280823

[5] Sangha V, Dhingra LS, Khera R. A multilabel deep learning model for the detection of conduction and rhythm disorders from PDF outputs of a widely available portable ECG device. Eur Heart J 2024;45:ehae666-3437.

[6] Khan S, Komal Q, Abdul Q, Khalid M, Somaan A, Moula G, et al Efficacy of AI models in detecting heart failure using ECG data: a systematic review and meta-analysis. Cureus 2025;17:e78683. 10.7759/cureus.78683 PMC11891813 40065863

[7] Glinge C, Jabbari R, Risgaard B, Lynge TH, Engstrøm T, Albert CM, et al Symptoms before sudden arrhythmic death syndrome: a nationwide study among the young in Denmark. J Cardiovasc Electrophysiol 2015;26:761–767. 10.1111/jce.12674 25807988

[8] Fiorina L, Chemaly P, Cellier J, Said MA, Coquard C, Younsi S, et al Artificial intelligence–based electrocardiogram analysis improves atrial arrhythmia detection from a smartwatch electrocardiogram. Eur Heart J Digit Health 2024;5:535–541. 10.1093/ehjdh/ztae047 PMC11417483 39318690

[9] Reljin N, Lazaro J, Hossain MB, Noh YS, Cho CH, Chon KH. Using the redundant convolutional encoder–decoder to denoise QRS complexes in ECG signals recorded with an armband wearable device. Sensors 2020;20:4611. 10.3390/s20164611 PMC7472132 32824420

[10] Soares QB, Andrade DA, Ribeiro E, Verardino RG, Reis TC, Samesima N, et al Clinical assessment of a lightweight cnn model for real-time atrial fibrillation prediction in continuous wearable monitoring, In: 2024 46th Annual International Conference of the IEEE Engineering in Medicine and Biology Society (EMBC): Piscataway, NJ, USA: IEEE; 2024. p1–4. 10.1109/EMBC53108.2024.10781856 40039447

[11] Mannhart D, Lefebvre B, Gardella C, Henry C, Serban T, Knecht S, et al Clinical validation of an artificial intelligence algorithm offering cross-platform detection of atrial fibrillation using smart device electrocardiograms. Arch Cardiovasc Dis 2023;116:249–257. 10.1016/j.acvd.2023.04.003 37183163

[12] Hannun AY, Rajpurkar P, Haghpanahi M, Tison GH, Bourn C, Turakhia MP, et al Cardiologist-level arrhythmia detection and classification in ambulatory electrocardiograms using a deep neural network. Nat Med 2019;25:65–69. 10.1038/s41591-018-0268-3 PMC6784839 30617320

[13] Halvaei H, Svennberg E, Sörnmo L, Stridh M. False alarm reduction in atrial fibrillation screening, In: 2020 Computing in Cardiology: Rimini, Italy: IEEE; 2020. p1–4.

[14] An X, Liu Y, Zhao Y, Lu S, Stylios GK, Liu Q. Adaptive motion artifact reduction in wearable ECG measurements using impedance pneumography signal. Sensors 2022;22:5493. 10.3390/s22155493 PMC9332869 35897997

[15] Castaño Usuga FA, Gissel C, Hernández AM. Motion artifact reduction in electrocardiogram signals through a redundant denoising independent component analysis method for wearable health care monitoring systems: algorithm development and validation. JMIR Med Inform 2022;10:e40826. 10.2196/40826 PMC9736764 36274196

[16] Rajakariar K, Koshy AN, Sajeev JK, Nair S, Roberts L, Teh AW. Accuracy of a smartwatch based single-lead electrocardiogram device in detection of atrial fibrillation. Heart 2020;106:665–670. 10.1136/heartjnl-2019-316004 31911507

[17] Salvi M, Acharya MR, Seoni S, Faust O, Tan RS, Barua PD, et al Artificial intelligence for atrial fibrillation detection, prediction, and treatment: a systematic review of the last decade (2013–2023). Wiley Interdiscip Rev Data Min Knowl Discov 2024;14:e1530.

[18] Wang R, Veera SC, Asan O, Liao T. A systematic review on the use of consumer-based ECG wearables on cardiac health monitoring. IEEE J Biomed Health Inform 2024;28:6525–6537. 10.1109/JBHI.2024.3456028 39240746

[19] Zepeda-Echavarria A, van de Leur RR, van Sleuwen M, Hassink RJ, Wildbergh TX, Doevendans PA, et al Electrocardiogram devices for home use: technological and clinical scoping review. JMIR cardio 2023;7:e44003. 10.2196/44003 PMC10362423 37418308

[20] Giustini DM . Undermind.ai (product review). J Can Health Libr Assoc 2025;46:42–46.

[21] Hunter R, Booth A, Wood L. Searching smarter, not harder: leveraging AI to enhance literature searches for theory-driven reviews—a methodological case study. BMC Med Res Methodol 2026;26:82. 10.1186/s12874-026-02814-3 PMC13067685 41781892

[22] Yang Z, Wang C. A U-net-based deep learning method for R-wave detection in wearable device, eds. 2024 3rd International Conference on Electronics and Information Technology (EIT) 2024 Sep 20–22; Chengdu, China: Piscataway, NJ, USA: IEEE; 2024. p 67–70.

[23] Kwon JM, Jo YY, Lee SY, Kang S, Lim SY, Lee MS, et al Artificial intelligence-enhanced smartwatch ECG for heart failure-reduced ejection fraction detection by generating 12-lead ECG. Diagnostics 2022;12:654. 10.3390/diagnostics12030654 PMC8947562 35328207

[24] Hammer A, Goettling M, Malberg H, Linke A, Schmidt M. An explainable AI for trustworthy detection of atrial fibrillation on reduced lead ECGs in mobile applications. Eur Heart J 2024;45:ehae666-3497.

[25] Lueken M, Mettner J, Spicher N, Gramlich M, Marx N, Leonhardt S, et al Towards artificial intelligence-based decision support for large-scale screening for atrial fibrillation. IEEE J Biomed Health Inform 2025;29:7633–7642. 10.1109/JBHI.2025.3579621 40512642

[26] Prasad VK, Raj KN, Muthya S, Nair RS. Development and validation of ECG rhythm classification on a multitude of data sources using deep learning, In: 2021 IEEE 9th International Conference on Healthcare Informatics (ICHI); 2021 Aug 9–12; Victoria, BC, Canada. Piscataway, NJ, USA: IEEE; 2021. p101–105.

[27] Patlatzoglou K, Pastika L, Barker J, Sieliwonczyk E, Khattak GR, Zeidaabadi B, et al The cost of explainability in artificial intelligence-enhanced electrocardiogram models. NPJ Digit Med 2025;8:747. 10.1038/s41746-025-02122-y PMC12680752 41350577

[28] Ding EY, Han D, Whitcomb C, Bashar SK, Adaramola O, Soni A, et al Accuracy and usability of a novel algorithm for detection of irregular pulse using a smartwatch among older adults: observational study. JMIR cardio 2019;3:e13850. 10.2196/13850 PMC6834225 31758787

[29] Wan E, Glotzer T, Mittal S, Senepart O, Lefebvre B. Artificial intelligence-based improvement of smartwatch detection of AF. Circulation 2024;150:A4147060.

[30] Iqhrammullah M, Abdullah A, Hermansyah, Ichwansyah F, Rani HA, Alina M, et al Accuracy and interpretability of smartwatch electrocardiogram for early detection of atrial fibrillation: a systematic review and meta-analysis. J Arrhythm 2025;41:e70087. 10.1002/joa3.70087 PMC12096014 40406413

[31] Xie C, Wang Z, Yang C, Liu J, Liang H. Machine learning for detecting atrial fibrillation from ECGs: systematic review and meta-analysis. Rev Cardiovasc Med 2024;25:8. 10.31083/j.rcm2501008 PMC11262392 39077651

[32] Choi JH, Song SH, Kim J, Jeon J, Woo K, Cho SJ, et al Machine learning algorithms for predicting atrial fibrillation using single-lead data derived from 12-lead ECGs. Front Cardiovasc Med 2025;12:1612750. 10.3389/fcvm.2025.1612750 PMC12537692 41126917

[33] Hamami H, Solewicz Y, Zur D, Kleerekoper Y, Behar JA. UPVC-Net: a universal premature ventricular contraction detection deep learning algorithm. IEEE Trans Biomed Eng 2026;73:3567–3575. 10.1109/TBME.2026.3655531 41557574

[34] Yildirim Ö . A novel wavelet sequence based on deep bidirectional LSTM network model for ECG signal classification. Comput Biol Med 2018;96:189–202. 10.1016/j.compbiomed.2018.03.016 29614430

[35] Acharya UR, Oh SL, Hagiwara Y, Tan JH, Adam M, Gertych A, et al A deep convolutional neural network model to classify heartbeats. Comput Biol Med 2017;89:389–396. 10.1016/j.compbiomed.2017.08.022 28869899

[36] Romdhane TF, Pr MA. Electrocardiogram heartbeat classification based on a deep convolutional neural network and focal loss. Comput Biol Med 2020;123:103866. 10.1016/j.compbiomed.2020.103866 32658786

[37] Cho J, Lee B, Kwon JM, Lee Y, Park H, Oh BH, et al Artificial intelligence algorithm for screening heart failure with reduced ejection fraction using electrocardiography. ASAIO J 2021;67:314–321. 10.1097/MAT.0000000000001218 33627606

[38] Attia ZI, Harmon DM, Dugan J, Manka L, Lopez-Jimenez F, Lerman A, et al Prospective evaluation of smartwatch-enabled detection of left ventricular dysfunction. Nat Med 2022;28:2497–2503. 10.1038/s41591-022-02053-1 PMC9805528 36376461

[39] Bachtiger P, Petri CF, Scott FE, Park SR, Kelshiker MA, Sahemey HK, et al Point-of-care screening for heart failure with reduced ejection fraction using artificial intelligence during ECG-enabled stethoscope examination in London, UK: a prospective, observational, multicentre study. Lancet Digit Health 2022;4:e117–e125. 10.1016/S2589-7500(21)00256-9 PMC8789562 34998740

[40] Guo L, Pressman GS, Kieu SN, Marrus SB, Mathew G, Prince J, et al Automated detection of reduced ejection fraction using an ECG-enabled digital stethoscope: a large cohort validation. JACC Adv 2025;4:101619. 10.1016/j.jacadv.2025.101619 PMC11891699 39983614

[41] Alam R, Aguirre A, Stultz CM. Detecting QT prolongation from a single-lead ECG with deep learning. PLOS Digit Health 2024;3:e0000539. 10.1371/journal.pdig.0000539 PMC11198807 38917157

[42] Angelaki E, Barmparis GD, Fragkiadakis K, Maragkoudakis S, Zacharis E, Plevritaki A, et al Diagnostic performance of single-lead electrocardiograms for arterial hypertension diagnosis: a machine learning approach. J Hum Hypertens 2025;39:58–65. 10.1038/s41371-024-00969-4 39424986

[43] Sau A, Pastika L, Sieliwonczyk E, Patlatzoglou K, Ribeiro AH, McGurk KA, et al Artificial intelligence-enabled electrocardiogram for mortality and cardiovascular risk estimation: a model development and validation study. Lancet Digit Health 2024;6:e791–e802. 10.1016/S2589-7500(24)00172-9 39455192

[44] Lin C, Liu WT, Chen KC, Tsai DJ, Chang DW, Tsai TN, et al Bridging the gap from clinical to home ECG: quantifying and overcoming accuracy loss in AI-enabled single-lead ECG models. NPJ Digit Med 2026;9:510. 10.1038/s41746-026-02919-5 PMC13338164 42410026

[45] Halvaei H, Svennberg E, Sörnmo L, Stridh M. Identification of transient noise to reduce false detections in screening for atrial fibrillation. Front Physiol 2021;12:672875. 10.3389/fphys.2021.672875 PMC8212862 34149452

[46] Perez MV, Mahaffey KW, Hedlin H, Rumsfeld JS, Garcia A, Ferris T, et al Large-scale assessment of a smartwatch to identify atrial fibrillation. N Engl J Med 2019;381:1909–1917. 10.1056/NEJMoa1901183 PMC8112605 31722151

[47] Lubitz SA, Faranesh AZ, Selvaggi C, Atlas SJ, McManus DD, Singer DE, et al Detection of atrial fibrillation in a large population using wearable devices: the fitbit heart study. Circulation 2022;146:1415–1424. 10.1161/CIRCULATIONAHA.122.060291 PMC9640290 36148649

[48] Petek BJ, Al-Alusi MA, Moulson N, Grant AJ, Besson C, Guseh JS, et al Consumer wearable health and fitness technology in cardiovascular medicine: JACC state-of-the-art review. J Am Coll Cardiol 2023;82:245–264. 10.1016/j.jacc.2023.04.054 PMC10662962 37438010

[49] Lawal L, Paton C, English M, Holthof B, Preston T. Mapping algorithmic bias in AI-powered electrocardiogram interpretation across the AI life cycle: protocol for a scoping review. JMIR Res Protoc 2026;15:e82486. 10.2196/82486 PMC12869145 41556569

[50] Dhingra LS, Aminorroaya A, Oikonomou EK, Nargesi AA, Wilson FP, Krumholz HM, et al Use of wearable devices in individuals with or at risk for cardiovascular disease in the US, 2019 to 2020. JAMA Netw Open 2023;6:e2316634. 10.1001/jamanetworkopen.2023.16634 PMC10248745 37285157

[51] Huhn S, Matzke I, Koch M, Gunga HC, Maggioni MA, Sié A, et al Using wearable devices to generate real-world, individual-level data in rural, low-resource contexts in Burkina Faso, Africa: a case study. Front Public Health 2022;10:972177. 10.3389/fpubh.2022.972177 PMC9561896 36249225

[52] Noseworthy PA, Attia ZI, Brewer LC, Hayes SN, Yao X, Kapa S, et al Assessing and mitigating bias in medical artificial intelligence: the effects of race and ethnicity on a deep learning model for ECG analysis. Circ Arrhythm Electrophysiol 2020;13:e007988. 10.1161/CIRCEP.119.007988 PMC7158877 32064914

[53] Mihan A, Pandey A, Van Spall HG. Artificial intelligence bias in the prediction and detection of cardiovascular disease. NPJ Cardiovasc Health 2024;1:31. 10.1038/s44325-024-00031-9 PMC12912404 41775892

[54] Moons KG, Damen JA, Kaul T, Hooft L, Navarro CA, Dhiman P, et al PROBAST+ AI: an updated quality, risk of bias, and applicability assessment tool for prediction models using regression or artificial intelligence methods. bmj 2025;388:e082505. 10.1136/bmj-2024-082505 PMC11931409 40127903

[55] Liu X, Rivera SC, Moher D, Calvert MJ, Denniston AK, Ashrafian H, et al Reporting guidelines for clinical trial reports for interventions involving artificial intelligence: the CONSORT-AI extension. Lancet Digit Health 2020;2:e537–e548. 10.1016/S2589-7500(20)30218-1 PMC8183333 33328048

[56] Collins GS, Moons KG, Dhiman P, Riley RD, Beam AL, Van Calster B, et al TRIPOD+ AI statement: updated guidance for reporting clinical prediction models that use regression or machine learning methods. bmj 2024;385:q902. 10.1136/bmj-2023-078378 PMC11019967 38626948

[57] Foryciarz A, Pfohl SR, Patel B, Shah N. Evaluating algorithmic fairness in the presence of clinical guidelines: the case of atherosclerotic cardiovascular disease risk estimation. BMJ Health Care Inform 2022;29:e100460. 10.1136/bmjhci-2021-100460 PMC8996004 35396247

[58] Pfohl SR, Foryciarz A, Shah NH. An empirical characterization of fair machine learning for clinical risk prediction. J Biomed Inform 2021;113:103621. 10.1016/j.jbi.2020.103621 PMC7871979 33220494

[59] Chen IY, Szolovits P, Ghassemi M. Can AI help reduce disparities in general medical and mental health care? . AMA J Ethics 2019;21:167–179. 10.1001/amajethics.2019.167 30794127

[60] Hughes A, Shandhi MM, Master H, Dunn J, Brittain E. Wearable devices in cardiovascular medicine. Circ Res 2023;132:652–670. 10.1161/CIRCRESAHA.122.322389 PMC9991078 36862812

[61] Chen W, Khurshid S, Singer DE, Atlas SJ, Ashburner JM, Ellinor PT, et al Cost-effectiveness of screening for atrial fibrillation using wearable devices. JAMA Health Forum 2022;3:e222419–e222419. 10.1001/jamahealthforum.2022.2419 PMC9356321 36003419

[62] Tran HH, Thu A, Fuertes A, Twayana AR, Mahadevaiah A, Mehta KA, et al Electrocardiogram-based artificial intelligence for detection of low ejection fraction: a contemporary review. Cardiol Rev 2025:10–97. 10.1097/CRD.0000000000000975 40548850

[63] Gala DA, Sharp DA, Schramm MD, Ries DW, Pope DM, Leo DM, et al Single-lead ECGs with wearable technology: diagnostic accuracy in patients with cardiovascular disease. Europace 2023;25:euad122-619.

[64] Isenegger C, Mannhart D, Weidlich S, Arnet R, Du Fay De Lavallaz J, Krisai P, et al Diagnostic accuracy of smartwatches for automated atrial fibrillation detection over time: insights from the Basel wearable study. Europace 2024;26:euae102-681. 10.1016/j.jacep.2024.09.019 39545917

[65] Wyatt KD, Poole LR, Mullan AF, Kopecky SL, Heaton HA. Clinical evaluation and diagnostic yield following evaluation of abnormal pulse detected using apple watch. J Am Med Inform Assoc 2020;27:1359–1363. 10.1093/jamia/ocaa137 PMC7526465 32979046

[66] Tran KV, Filippaios A, Noorishirazi K, Ding E, Han D, Mohagheghian F, et al False atrial fibrillation alerts from smartwatches are associated with decreased perceived physical well-being and confidence in chronic symptoms management. Cardiol Cardiovasc Med 2023;7:97. 10.26502/fccm.92920314 PMC10358285 37476150

[67] Rosman L, Lampert R, Zhuo S, Li Q, Varma N, Burg M, et al Wearable devices, health care use, and psychological well-being in patients with atrial fibrillation. J Am Heart Assoc 2024;13:e033750. 10.1161/JAHA.123.033750 PMC11964026 39011944

[68] O’Shea CJ, Middeldorp ME, Hendriks JM, Brooks AG, Lau DH, Emami M, et al Remote monitoring alert burden: an analysis of transmission in> 26,000 patients. JACC Clin Electrophysiol 2021;7:226–234. 10.1016/j.jacep.2020.08.029 33602404

[69] O’Shea CJ, Middeldorp ME, Hendriks JM, Brooks AG, Harper C, Thomas G, et al Remote monitoring of implantable loop recorders: false-positive alert episode burden. Circ Arrhythm Electrophysiol 2021;14:e009635. 10.1161/CIRCEP.121.009635 34708660

[70] Covino S, Russo V. False-positive alarms in patients with implantable loop recorder followed by remote monitoring: a systematic review. Pacing Clin Electrophysiol 2024;47:406–416. 10.1111/pace.14941 38341627

[71] Afzal MR, Mease J, Koppert T, Okabe T, Tyler J, Houmsse M, et al Incidence of false-positive transmissions during remote rhythm monitoring with implantable loop recorders. Heart Rhythm 2020;17:75–80. 10.1016/j.hrthm.2019.07.015 31323348

[72] Neiman ZM, Raitt MH, Rohrbach G, Dhruva SS. Monitoring of remotely reprogrammable implantable loop recorders with algorithms to reduce false-positive alerts. J Am Heart Assoc 2024;13:e032890. 10.1161/JAHA.123.032890 PMC10944033 38390808

[73] Middeldorp ME, Thomas G, Kamsani SH, Harper C, Baykaner T, Gopinathannair R, et al Use of artificial intelligence algorithms to reduce transmissions in implantable loop recorders. Heart rhythm 2025;22:e241–e243. 10.1016/j.hrthm.2024.10.024 PMC12006446 39427689

[74] Katapadi A, Chelikam N, Rosemas S, Higuera L, Colombowala I, Bansal S, et al Impact of artificial intelligence-enhanced insertable cardiac monitors on device clinic workflow and resource utilization. JACC Adv 2025;4:101656. 10.1016/j.jacadv.2025.101656 PMC11968260 40107046

[75] Bisignani G, Cheung JW, Rordorf R, Kutyifa V, Hofer D, Berti D, et al Implantable cardiac monitors: artificial intelligence and signal processing reduce remote ECG review workload and preserve arrhythmia detection sensitivity. Front Cardiovasc Med 2024;11:1343424. 10.3389/fcvm.2024.1343424 PMC10844377 38322767

[76] Quartieri F, Marina-Breysse M, Pollastrelli A, Paini I, Lizcano C, Lillo-Castellano JM, et al Artificial intelligence augments detection accuracy of cardiac insertable cardiac monitors: results from a pilot prospective observational study. Cardiovasc Digit Health J 2022;3:201–211. 10.1016/j.cvdhj.2022.07.071 PMC9596320 36310681

[77] Ferrick AM, Raj SR, Deneke T, Kojodjojo P, Lopez-Cabanillas N, Abe H, et al 2023 HRS/EHRA/APHRS/LAHRS expert consensus statement on practical management of the remote device clinic. Europace 2023;25:euad123. 10.1093/europace/euad123 PMC10199172 37208301

[78] Kelly CJ, Karthikesalingam A, Suleyman M, Corrado G, King D. Key challenges for delivering clinical impact with artificial intelligence. BMC Med 2019;17:195. 10.1186/s12916-019-1426-2 PMC6821018 31665002

[79] U.S. Food and Drug Administration . Marketing Submission Recommendations for a Predetermined Change Control Plan for Artificial Intelligence-Enabled Device Software Functions: Guidance for Industry and Food and Drug Administration Staff. Silver Spring, MD, USA: U.S. Food and Drug Administration; 2025. Issued August 18, 2025. Accessed October 9, 2026.

[80] Wei Q, Xu G, Liu Y. Comparative study of international medical AI regulatory policies: regional approaches and evidence from clinical practice. AI Soc 2026;41:6089–6123.

[81] Medical Device Coordination Group . Medical Device Coordination Group; Artificial Intelligence Board. MDCG 2025-6/AIB 2025-1: FAQ on Interplay Between the Medical Devices Regulation (MDR) & In Vitro Diagnostic Medical Devices Regulation (IVDR) and the Artificial Intelligence Act (AIA). Brussels, Belgium: European Commission; 2025. Published June 2025: European Commission; 2025.

[82] Medical Device Coordination Group . Medical Device Coordination Group. MDCG 2023-4: Medical Device Software (MDSW) – Hardware Combinations: Guidance on MDSW Intended to Work in Combination with Hardware or Hardware Components. Brussels, Belgium: European Commission; 2023. Published October 18, 2023.: European Commission; 2023.

[83] Hughes-Noehrer L, Channer L, Strain G, Yates G, Body R, Jay C. Contrasting attitudes towards current and future artificial intelligence applications for computerised interpretation of electrocardiograms: a clinical stakeholder interview study. JAMIA Open 2025;8:ooaf071. 10.1093/jamiaopen/ooaf071 PMC12278055 40692805

[84] Amann J, Blasimme A, Vayena E, Frey D, Madai VI; Precise4Q Consortium. Explainability for artificial intelligence in healthcare: a multidisciplinary perspective. BMC Med Inform Decis Mak 2020;20:310. 10.1186/s12911-020-01332-6 PMC7706019 33256715

[85] Toparti O, Rajput K, Darzi A, Ghafur S. Cybersecurity in connected medical devices: a policy agenda for the NHS. NPJ Digit Med 2026;9:204. 10.1038/s41746-026-02534-4 PMC12976036 41807747

